# Comparative Genomics of *Pseudomonas* sp. Strain SI-3 Associated With Macroalga *Ulva prolifera*, the Causative Species for Green Tide in the Yellow Sea

**DOI:** 10.3389/fmicb.2018.01458

**Published:** 2018-07-02

**Authors:** Huihui Fu, Peng Jiang, Jin Zhao, Chunhui Wu

**Affiliations:** ^1^CAS Key Laboratory of Experimental Marine Biology, Institute of Oceanology, Chinese Academy of Sciences, Qingdao, China; ^2^Laboratory for Marine Biology and Biotechnology, Qingdao National Laboratory for Marine Science and Technology, Qingdao, China; ^3^Center for Ocean Mega-Science, Chinese Academy of Sciences, Qingdao, China

**Keywords:** *Pseudomonas*, genome sequence, Comparative genomics, *Ulva prolifera*, algae-associated bacteria

## Abstract

Algae-bacteria associations occurred widely in marine habitats, however, contributions of bacteria to macroalgal blooming were almost unknown. In this study, a potential endophytic strain SI-3 was isolated from *Ulva prolifera*, the causative species for the world's largest green tide in the Yellow Sea, following a strict bleaching treatment to eliminate epiphytes. The genomic sequence of SI-3 was determined in size of 4.8 Mb and SI-3 was found to be mostly closed to *Pseudomonas stutzeri*. To evaluate the characteristics of SI-3 as a potential endophyte, the genomes of SI-3 and other 20 *P. stutzeri* strains were compared. We found that SI-3 had more strain-specific genes than most of the 20 *P. stutzeri* strains. Clusters of Orthologous Groups (COGs) analysis revealed that SI-3 had a higher proportion of genes assigned to transcriptional regulation and signal transduction compared with the 20 *P. stutzeri* strains, including four rhizosphere bacteria, indicating a complicated interaction network between SI-3 and its host. *P. stutzeri* is renowned for its metabolic versatility in aromatic compounds degradation. However, significant gene loss was observed in several aromatic compounds degradation pathways in SI-3, which may be an evolutional adaptation that developed upon association with its host. KEGG analysis revealed that dissimilatory nitrate reduction to ammonium (DNRA) and denitrification, two competing dissimilatory nitrate reduction pathways, co-occurred in the genome of SI-3, like most of the other 20 *P. stutzeri* strains. We speculated that DNRA of SI-3 may contribute a competitive advantage in nitrogen acquisition of *U. prolifera* by conserving nitrogen in ${\text{NH}}_{4}^{+}$ form, as in the case of microalgae bloom. Collectively, these data suggest that *Pseudomonas* sp. strain SI-3 was a suitable candidate for investigation of the algae-bacteria interaction with *U. prolifera* and the ecological impacts on algal blooming.

## Introduction

Substance exchange is considered the fundation of the partnership between algae and associated bacteria (Dittami et al., 2014; Cooper and Smith, 2015; Kouzuma and Watanabe, 2015). Algae, as the primary producer in freshwater and marine environments, provide dissolved organic nutrients to phycosphere heterotrophic bacteria (Field et al., 1998; Kouzuma and Watanabe, 2015). Complementarily, associated bacteria contribute to growth, morphogenesis, spore germination and colonization of algae by fixing nitrogen, as well as releasing minerals, vitamins, auxins, and quorum sensing signaling molecules (Joint et al., 2002; Croft et al., 2005; Marshall et al., 2006; Weinberger et al., 2007; Goecke et al., 2010; Foster et al., 2011; Singh and Reddy, 2014, 2015). Algae-associated bacteria have the potential to stimulate the growth of algae, similar to plant growth-promoting rhizobacteria. In addition, algae-associated bacteria can confer the competitive advantages to their host. Although limited in study, it has been suggested that associated bacteria promote algae bloom in the interaction between diatom and bacteria (Doucette, 1995).

Most, if not all, algae live with associated bacteria (Delbridge et al., 2004; Dittami et al., 2014). It generally accepted that algae exudates, such as polysaccharides, amino acids, proteoglycans or glycoproteins, form a phycosphere and influence the community structure of algae-associated bacteria (Myklestad, 1995; Sapp et al., 2007; Sison-Mangus et al., 2014). 16S rDNA sequencing and denaturing gradient gel electrophoresis (DGGE) fingerprinting have revealed that algae-associated bacteria communities are highly distinct from those of planktonic communities (Burke et al., 2011a; Amin et al., 2012; Goecke et al., 2013). Furthermore, algae-associated bacteria community presents host specificity. For example, pyrosequencing of 16S rDNA genes revealed that three species of the diatom genus *Pseudo-nitzschia* have different bacterial community compositions (Sison-Mangus et al., 2014). According to the spatial distribution of algae-associated bacteria, they can be divided into epiphyte and endophyte. Burke et al. (2011b) revealed the epiphytic bacterial community structure of *Ulva australis* varied according to space and time. However, the endophytic bacteria of algae are more closely associated withtheir host. For example, the endophytic bacterial communities of *Bryopsis* were found to be well-defined, even though samples were collected several hundred kilometers apart (Hollants et al., 2011). Additionally, the relative stability of endophytic bacterial communities of algae was successfully used to trace the invasive *Caulerpa racemosa* in Mediterranean to Australian range (Aires et al., 2013).

*Ulva prolifera* is the only dominant alga that causes successive green tides in the Yellow Sea, China (Zhao et al., 2013; Li et al., 2016), inducing harmful ecological impacts and economic losses. The community structures of *U. prolifera* associated bacteria have also been investigated to obtain information regarding the cause and influence of the world's largest green tide, but these studies were limited to communities from thalli surface and environmental water during the algal blooming (Guo et al., 2011; Liu et al., 2011). Moreover, the endophytic bacteria of *U. prolifera* have never been studied. In the present study, we obtained potential endophytes by treating the thalli of *U. prolifera* with ethanol plus bleach for sterilization. This method has been extensively used to eliminate epiphytes of algae and higher plants (Coombs and Franco, 2003; Kientz et al., 2011; Aires et al., 2012; Baoune et al., 2018). *Pseudomonas* sp. strain SI-3, which is mostly closed to *P. stutzeri*, was isolated from rich medium in which the homogenate of pretreated thalli was plated, indicating that strain SI-3 was probably an endophyte of *U. prolifera*.

*Pseudomonas* is a diverse genus that is known to occupy a wide range of niches and metabolic versatility. As a remarkable member of the *Pseudomonas* genus, *P. stutzeri* has received particular attention for its ability to conduct denitrification, degradation of aromatic compounds, and nitrogen-fixation (Lalucat et al., 2006). Some *P. stutzeri* strains even associate with plants endophytically or in the rhizosphere, stimulating plant growth or protecting plants against pathogens (Yan et al., 2008; Shen et al., 2013). In this study, the genome of strain SI-3 was sequenced and compared with that of 20 other *P. stutzeri* strains from diverse environments. Comparative genomic analysis revealed distinct characteristics of strain SI-3. Overall, the availability of genome sequence of strain SI-3 and comparative genomics results suggest that *Pseudomonas* sp. strain SI-3 is a suitable candidate to further investigation of algae-bacteria interaction with seaweed host *U. prolifera*.

## Materials and methods

### Isolation and identification of *Pseudomonas* sp. strain SI-3

The protoplast of *U. prolifera* was prepared according to Wu et al. (accepted). Complete cells and protoplast of *U. prolifera* were observed by optical microscope to determine if endophytic bacteria exist. The external bacteria of thalli of *U. prolifera* were removed by ethanol plus bleach treatment according to Aires et al. (2012). Briefly, *U. prolifera* samples were placed in 99% ethanol for 1 min and transferred to 3% bleach solution for 5 min, then immersed in 99% ethanol for about 30 s. The effect of epiphytes removal of the treated sample was detected by scanning electron microscopy (SEM, S-3400N, Hitachi, Tokyo, Japan) using untreated sample as a control. The endophytic bacteria of *U. prolifera* were released by grinding thoroughly, then cultured on 2216E agar medium and cultured at 28^o^C. For identification, PCR amplification was conducted using universal bacterial 16S rRNA primers 8F and 1492R. Strain SI-3, which was identified by 16S rDNA sequencing to be most closely related to *P. stutzeri*, was used for genome sequencing.

### Genome sequencing and characterization

The genome of strain SI-3 was sequenced using PacBio RS II system and assembled using HGAP assembler. rRNAs and tRNAs were predicted using Barranp 0.4.2 and tRNAscan-SE v1.3.1, respectively. Protein coding sequences were predicted using Glimmer 3.02 and annotated by BLASTp alignment (BLAST 2.2.28+) with the Non-redundant (Nr), string and GO databases. Gene islands were predicted using IslandPATH-DIMOB v1.0.0 and SIGI-HMM 4.0. Carbohydrate-active enzymes (CAZymes) were functional annotated using similarity searches against the CAZy database.

### Nucleotide sequence accession number

The complete nucleotide genome sequence of *Pseudomonas* sp. strain SI-3 has been deposited in GenBank under accession no. CP026511.

### 20 *P. stutzeri* strains for comparison with SI-3

We selected 20 *P. stutzeri* strains, including all 10 completely sequenced strains by far and 10 of 14 strains with genomes that were assembled into <100 contigs. The isolation environments of the 20 *P. stutzeri* strains were diverse, including seawater and marine sediment, rhizosphere, contaminated soil and water, and clinical specimens (Table 1) (Yan et al., 2008; Chen et al., 2011; Yu et al., 2011; Brunet-Galmés et al., 2012; Busquets et al., 2012, 2013; Li A. et al., 2012; Li X. et al., 2012; Chauhan et al., 2013; Grigoryeva et al., 2013; Smith et al., 2014; Hirose et al., 2015; Hu et al., 2015; Iyer and Damania, 2016; Wu et al., 2017).

**Table 1:** General information of *Pseudomonas* sp. strain SI-3 and 20 *P. stutzeri* strains.

**Strain**	**Genome (Mb)**	**Contigs**	**Accession no**.	**Characteristic of isolation site**
*Pseudomonas* sp. strain SI-3	4.84	1	CP026511	Endophyte of *Ulva prolifera*
*P. stutzeri* 28a24	4.73	1	CP007441.1	Soil, near the Tel Aviv airport
*P. stutzeri* ATCC 17588	4.55	1	CP002881.1	Clinical specimen
*P. stutzeri* A1501	4.57	1	CP000304.1	Rice paddy soil
*P. stutzeri* DSM4166	4.69	1	CP002622.1	Sorghum nutans cultivar rhizosphere
*P. stutzeri* CCUG 29243	4.71	1	CP003677.1	Polluted marine sediments
*P. stutzeri* DSM10701	4.17	1	CP003725.1	Soil
*P. stutzeri* RCH2	4.60	1	CP003071.1	Cr(VI) contaminated well
*P. stutzeri* 19SMN4	4.83	1	CP007509.1	Marine, Barcelona
*P. stutzeri* SLG510A3-8	4.65	1	CP011854.1	Oil-contaminatd soil
*P. stutzeri* 273	5.03	1	CP015641.1	Sediment of East China Sea
*P. stutzeri* KOS6	4.95	5	AMCZ00000000.2	Wastewaters from petrol industry factory
*P. stutzeri* NT0124	4.59	39	JXTL00000000.1	*Triticum turgidum* wheat rhizosphere
*P. stutzeri* TS44	4.28	78	AJXE00000000.1	Arsenic-contaminated soils of metal mine
*P. stutzeri* T13	4.65	71	ALJB00000000.1	Activated sludge from a wastewater treatment plant
*P. stutzeri* B1SMN1	5.32	78	AMVM00000000.1	Wastewater sample taken at a lagooning treatment plant
*P. stutzeri* MF28	4.94	91	ATAR00000000.1	Oyster-associated
*P. stutzeri* KF716	4.19	30	BBQQ00000000.1	Soil near a biphenyls manufacturing plant
*P. stutzeri* ODKF13	4.59	85	LSVE00000000.1	Soil farm from Alvin Texas
*P. stutzeri* AR9-4	4.58	18	MDGV00000000.1	Wild caught mosquito anopheles
*P. stutzeri* KMS-55	4.61	53	MUEH00000000.1	Rice root, India

### Comparative genomics

The genome sequence of SI-3 was compared with those of the 20 *P. stutzeri* above. Homologous genes were obtained by aligning the amino acids or nucleic acids sequences of all 21 strains using Ortho MCL v2.0.3 with an *E*-value <10^−5^. Conserved and strain-specific genes were identified by aligning the whole genome sequences using MUSCLE v3.7. The maximum likelihood (ML) phylogenetic tree based on the amino acids sequences of housekeeping gene *rpoD* which were aligned by MUSCLE v3.7, was constructed using MEGA 5. RAxML was used to construct the ML phylogenetic tree of concatenated amino acid sequences of single-copy orthologous conserved genes of 21 strains, after alignment using MUSCLE v3.7. For Clusters of Orthologous Groups (COGs) analysis, we used BLASTp (BLAST 2.2.28+) alignment against the string database (v9.05) to obtain the COG annotations of genes, then clustered them into different COG catagories. Metabolic pathway annotation was conducted using BLASTp (blastx/blastp 2.2.28+) against the Kyoto Encyclopedia of Genes and Genomes (KEGG) gene database.

## Results and discussion

### Isolation and genome features of *Pseudomonas* sp. strain SI-3

Seaweed-associated bacteria and their interactions with the hosts have attracted the attention of many researchers worldwide. Studies have revealed green macroalgae such as *Caulerpa* and *Bryopsis* contain endophytic bacteria (Dawes and Lohr, 1978; Delbridge et al., 2004; Hollants et al., 2011, 2013; Aires et al., 2013). However, the endophytic bacteria of *Ulva*, the cosmopolitan green macroalgae, remain poorly studied. We found numerous highly moving bacteria when observing the thalli of *U. prolifera* by optical microscope (see the Supplementary Video 1). In addition, the existence of highly moving bacteria was observed in the protoplast of *U. prolifera* using enzymatic digestion (Wu et al., accepted) as well by optical microscope (see the Supplementary Video 2). This is the first time the potential endophytic bacteria of *U. prolifera* have been visualized.

**Figure 1:**
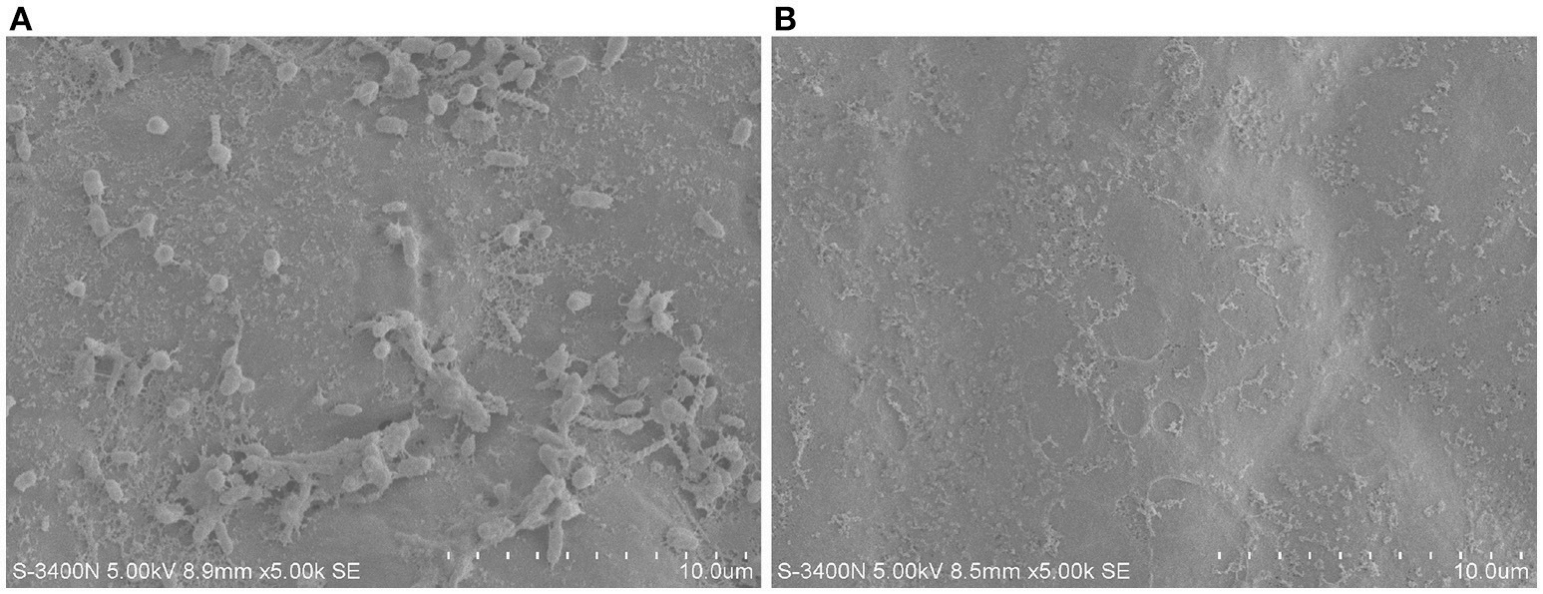
The epiphytes removal effect of ethanol plus bleach sterilization using SEM. **(A)** Untreated *U. prolifera*. **(B)** Ehtanol plus bleach treated *U. prolfiera*.

To isolate potential endophytic bacteria of *U. prolifera*, we eliminated the epiphytic bacteria using the ethanol plus bleach sterilization. In this combination, ethanol serves as a detergent/solvent to break down the phospholipid bilayer and promote the invasion of bleach. Moreover, bleach eliminates the epiphytes as a strong oxidant. This method has been used to efficiently eliminate epiphytic bacteria and chloroplastidial DNA of green alga *Caulerpa taxifolia* (Aires et al., 2012), remove epiphytic bacteria to extract bioactive compounds originating from macroalgae (Kientz et al., 2011), and isolate endophytic actinobacteria from roots of wheat and other plants grown in petroleum contaminated soil (Coombs and Franco, 2003; Baoune et al., 2018). The effect of epiphytes removal of *U. prolifera* was observed by SEM using the untreated sample as a control. No bacteria were observed on the surface of the treated sample (Figure 1), suggesting the efficient removal of epiphytic bacteria. This result intensively implied that the isolates we obtained from the treated thalli were endophytes.

The homogenate of treated thalli was cultured and a few of colonies appeared, among which some hard, dry, and coral-like colonies resembled *P. stutzeri* morphologically (Lalucat et al., 2006). Phylogenetic analysis of the 16S rDNA sequence of one isolate, designated as SI-3, showed 99% sequence identity to about 20 *P. stutzeri* strains. *P. stutzeri* species possess a high degree of genotypic heterogeneity, so they are easily confused during phylogenetic classification (Wolterink et al., 2002; Romanenko et al., 2005; Cladera et al., 2006; Lalucat et al., 2006). Since the systematically phylogenetic identification of strain SI-3 has not yet been completed, we identified it as *Pseudomonas* sp. strain SI-3, and this strain had been repeatedly obtained from bloomed *U. prolifera* samples in different years. *P. stutzeri* has received particular attention because of its metabolic versatility, such as the ability to fix nitrogen, as well as to conduct denitrification and degradation of aromatic compounds. In addition, some *P. stutzeri* strains form intimate relationships with plants. For example, *P. stutzeri* A1501 is capable of associating with or colonizing the higher plant and promotes its growth, and has been used as crop inoculants in China (You et al., 1995; Rediers et al., 2003).

**Figure 2:**
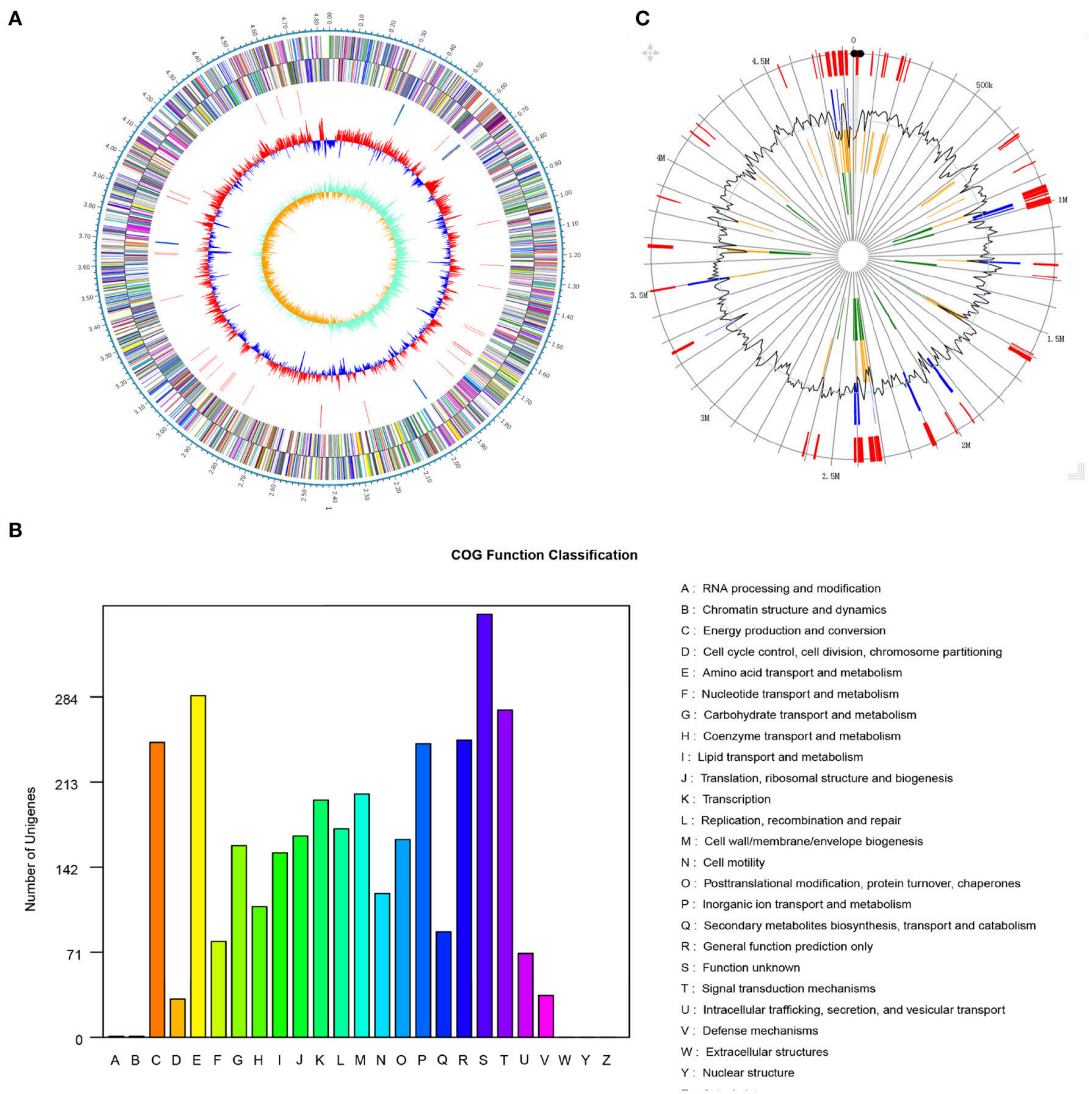
Genome features of *Pseudomonas* sp. strain SI-3. **(A)** Graphical map of chromosome of *Pseudomonas* sp. strain SI-3. The outer scale is marked in 0.1 Mb. From the outside to the center of each circle: Circle 1 and 2, genes encoded on forward and reverse strands, respectively. Coding sequences are colored by COG categories. Circle 3, RNA genes. Circle 4, GC content. Circle 5, GC skew (G–C/G+C). **(B)** COG function classification of SI-3. The ordinate axis indicates the gene numbers in each COG functional category. **(C)** Genomic islands predicted by two methods. Blue lines present the genomic islands predicted by IslandPATH-DIMOB method. Yellow lines present the genomic islands predicted by SIGI-HMM method. Red lines present the integrated genomic islands predicted by two methods. The second circle indicates the GC content.

The genome of *Pseudomonas* sp. strain SI-3 was sequenced using PacBio RS II system. A total 117394 reads were assembled into one single contig using HGAP assembler. SI-3 had a 60.22%-GC circular chromosome of 4,838.607 bp, with no extrachromosomal elements such as plasmid (Figure 2A). Nine rRNAs and 58 tRNAs were predicted using Barranp 0.4.2 and tRNAscan-SE v1.3.1, respectively. In total, 4,563 protein-coding sequences (CDSs) comprising approximately 88.1% of the genome were predicted using Glimmer 3.02. COG analysis assigned all genes into different functional categories, about 10% of which are functionally unknown (Figure 2B). Using IslandPATH-DIMOB v1.0.0 and SIGI-HMM 4.0, 68 genomic islands were predicted at least by one method in the genome of SI-3 (Figure 2C).

### General features of strain SI-3 genome compared with other *P. stutzeri* strains

#### Phylogeny

**Figure 3:**
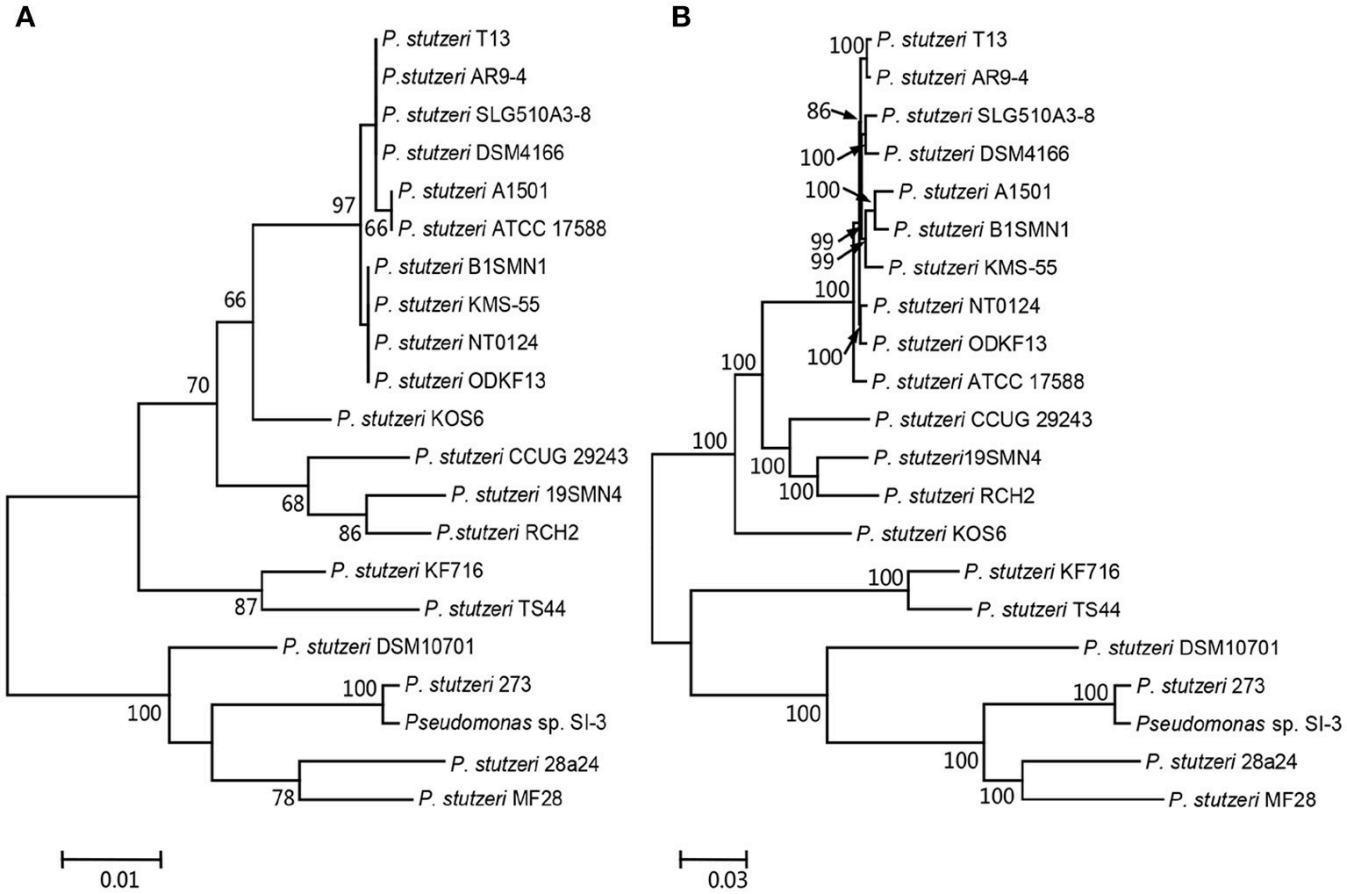
Phylogenetic trees of *Pseudomonas* sp. strain SI-3 and 20 other *P. stutzeri* strains. The Maximum Likelihood (ML) phylogeny trees were constructed based on amino acid sequences of housekeeping gene *rpoD* **(A)** and concatenated amino acid sequences of single copy orthologous genes **(B)**. The topology of the tree was tested with 1,000 bootstrap replications and bootstrap values were shown near the horizontal branches of the trees.

We compared SI-3 with the 20 *P. stutzeri* strains in Table 1, including all 10 strains that have been completely sequenced to dateand 10 of 14 strains for which their genomes were assembled into <100 contigs. The amino acid sequence of housekeeping gene *rpoD*, which is a qualified representative of the whole genomes for different *P. stutzeri* isolates (Yamamoto et al., 2000; Cladera et al., 2004) was selected as a genetic marker to construct a ML phylogenetic tree (Figure 3A). Moremover, a ML phylogenetic tree was established based on 2,291 concatenated amino acid sequences of single-copy orthologous genes from all strains (Figure 3B). Both trees shared a similar topological relationship and showed that SI-3 was mostly closed to *P. stutzeri* 273, which was isolated from sediment in a green-tide affected sea area (Wu et al., 2017), then clustered with 28a24 and MF28, which is a marine animal oyster-associated strain.

#### Pan- and core-genome

**Figure 4:**
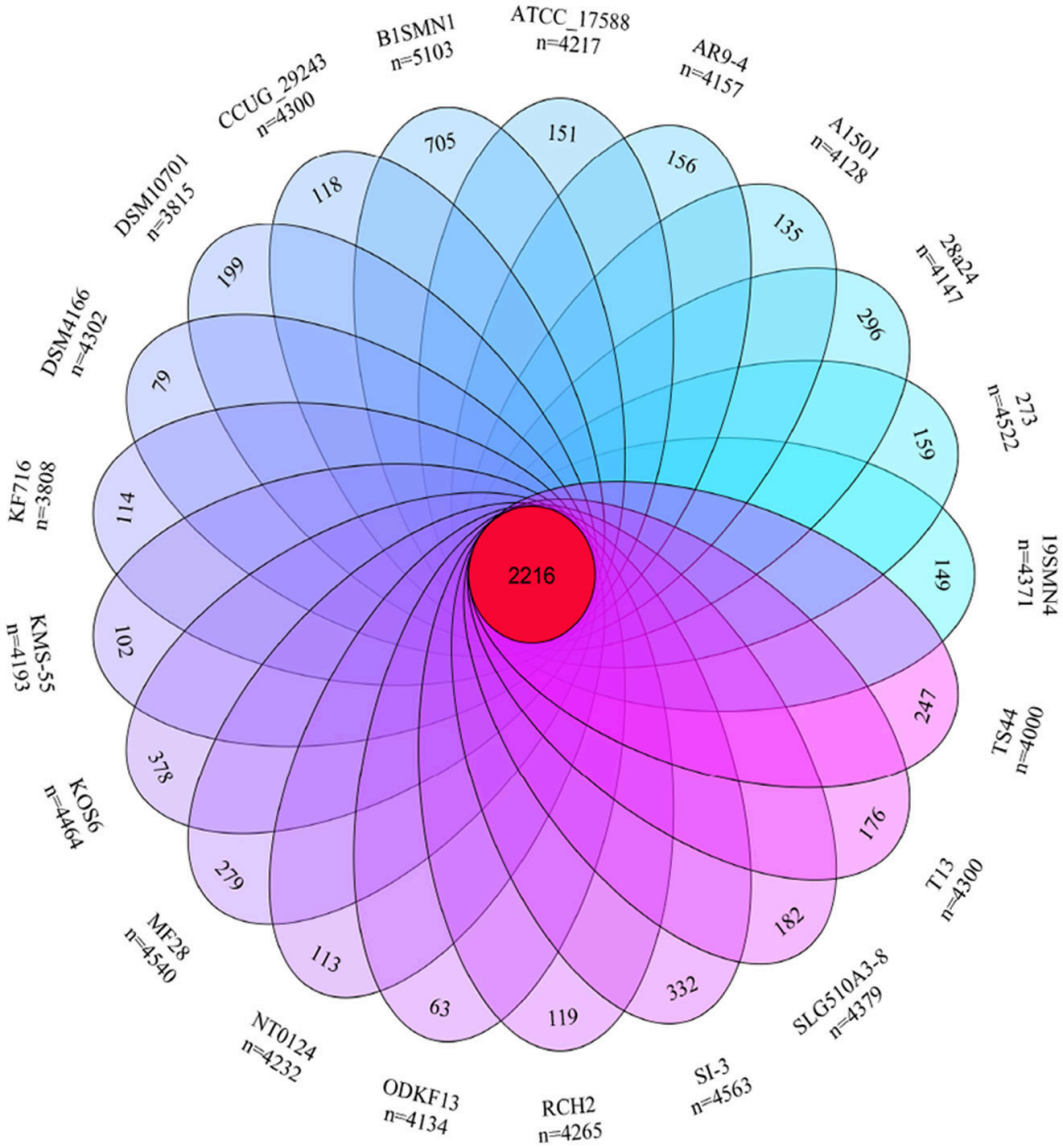
Venn diagram showing the pan- and core-genome of *Pseudomonas* sp. strain SI-3 and 20 *P. stutzeri* strains. The center red circle represented the core-genome. The strain name and total gene number of each ellipse presented were marked outside. The specific genes number of each strain was marked in corresponding ellipse.

The pan- and core-genome of strain SI-3 and the 20 *P. stutzeri* strains was analyzed to reveal unique genes of SI-3. We identified a pan-genome of 6,468 genes and a core-genome of 2,216 genes using 21 genomes (Figure 4). Taking into consideration of the average gene numbers of 4,282 for the 21 strains, the 2,216 genes of core-genome represent approximately 52% of the total genome. In other words, half of the genomic regions are conserved. *P. stutzeri* strains B1SMN1 and KOS6, and strain SI-3 were ranked highest based on the presence of 705, 378, and 332 strain-specific genes, respectively (Figure 4). For B1SMN1 and KOS6, which were both isolated from wastewater samples (Table 1), quite a few of their strain-specific genes encodes transporters, chemotaxis proteins, secretion systems, motility related proteins, and transcriptional regulators, which can contribute to the survival under extreme conditions. Thirteen of the strain-specific genes of strain SI-3, encoded kinases, transcriptional regulators, sensor proteins, chemotaxis proteins, and GGDEF-domain containing proteins, which are indicative of the need to respond to changes in the host environment (Burke et al., 2011a). Fifteen transposase genes are presented within or around the SI-3 specific genes regions, while 71 strain-specific genes are located in genomic islands, indicating the strain-specific genes of SI-3 may beexogenous. Moreover, up to 82% of the strain-specific genes of SI-3 are function-unknown, which may contribute to its survival and adaptation to unique environment.

### Enhanced transcription and signal transduction network

**Figure 5:**
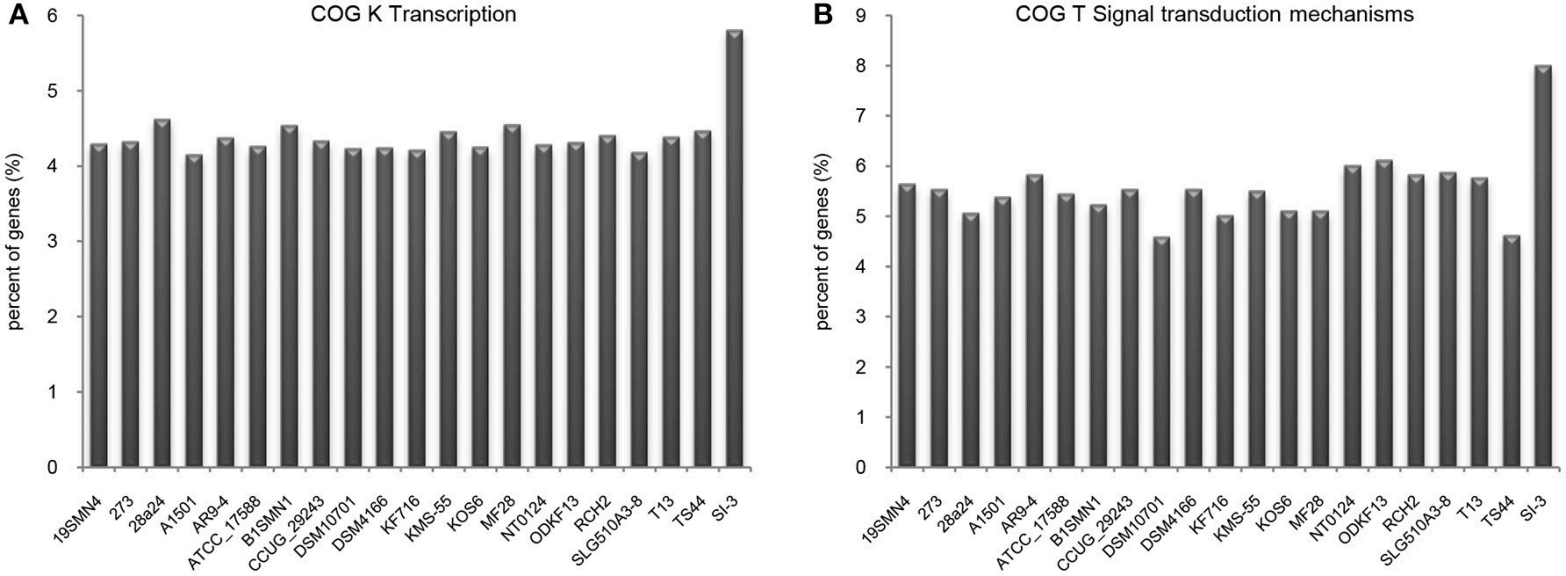
Enhanced transcription and signal transduction network in *Pseudomonas* sp. strain SI-3. The proportions of genes assigned into COG K **(A)** and COG T **(B)** categories were normalized to the total gene number of all COG categories in each strain.

We compared the functional distribution of all genes from 21 strains based on COG database. Strain SI-3 had 198 genes assigned to transcription (COG K) category, occupying 5.81% of the genes of all COG categories. This was the highest level among the 20 *P. stutzeri* strains, including the closely related strains 273 and 28a24 (Figure 5A). Transcriptional regulators were overrepresented in COG K category, which regulated transcription involved in attachment and colonization, quorum sensing, motility, and uptake of elements (Kovacikova and Skorupski, 1999; Cao et al., 2001; Fillat, 2014; Taw et al., 2015), indicating a complicated regulatory network within SI-3. In addition, genes enriched in signal transduction mechanism category (COG T) in SI-3 occupied 8.01% of the total COG categories genes, which was significantly higher than that of the other 20 *P. stutzeri* strains (Figure 5B). There were many homologs of the histidine kinase and GGDEF and EAL domain proteins, which were known to be involved in osmoregulation, chemotaxis, multidrug export, motility, and biofilm formation (Foynes et al., 2000; Nagakubo et al., 2002; Simm et al., 2004; Baker et al., 2006; Jenal and Malone, 2006; Yoshida et al., 2007), and these processes were involved in the colonization steps and interactions with other community members or the host. It is widely accepted that the host and its metabolites affect the gene regulation of associated bacteria (Mark et al., 2005; Pothier et al., 2007), and that the high proportion of transduction regulators and signal transducers could be indicative of the need of strain SI-3 to respond to changes in the host environment (Burke et al., 2011a). Notably, genes contributing to transcription and signal transduction mechanism in *P. stutzeri* strains A1501, DSM4166, NT0124, and KSM-55, which were isolated from rhizosphere of higher plants, were not as abundant as that in strain SI-3. We speculated that, as a potential endophytic bacterium, strain SI-3 may establish a more intimate and stable relationship with its host, than rhizosphere bacteria, therefore, they are prone to evolution of more complicated regulation and signal transduction networks to adapt to theire host environment. Collectively, these data indicate that strain SI-3 has evolved strain-specific features, which may contribute to its adaptability in the green macroalga *U. prolifera*.

### Loss of most aromatic compounds degradation genes

**Figure 6:**
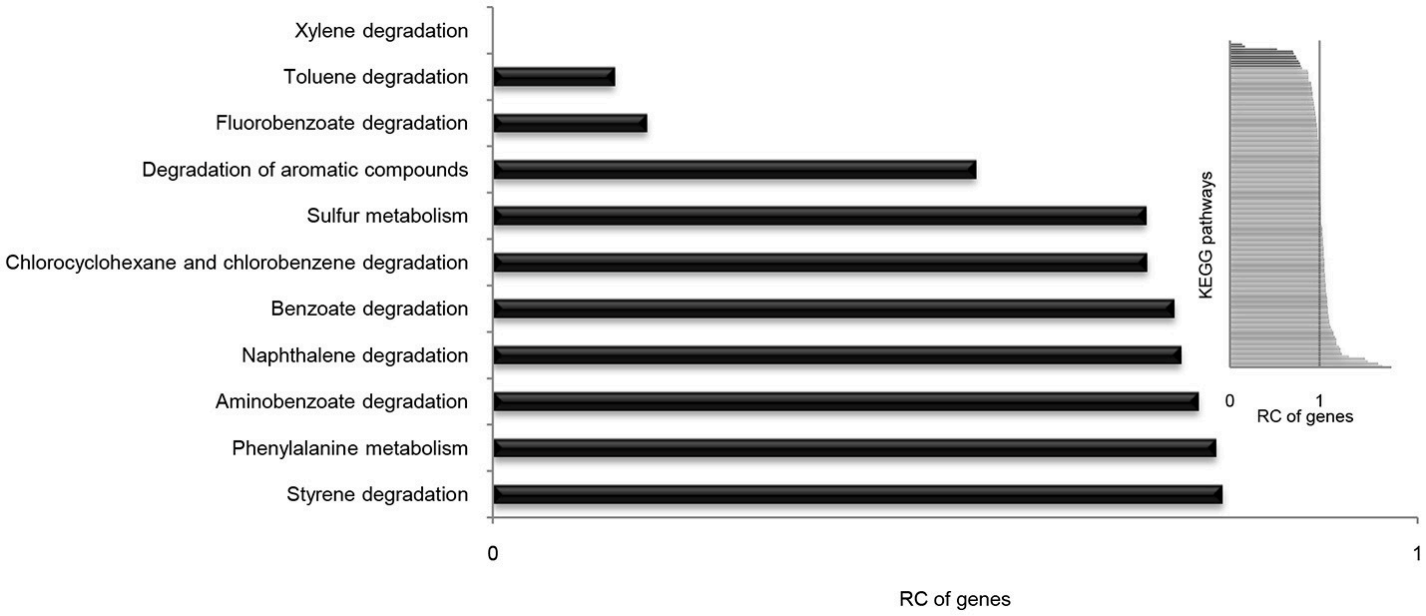
Relative contents (RCs) of gene numbers of KEGG metabolic pathways in *Pseudomonas* sp. strain SI-3. The average gene numbers for each metabolic pathway in strain SI-3 and 20 *P. stutzeri* strains were set to 1, after which the gene numbers of corresponding KEGG metabolic pathways of SI-3 were normalized. All pathways of SI-3 were ranked based on the RC of genes. The pathways with RCs between 0.8 and 1.2 were omitted to make the results clear. The embedded panel in the upper right was the entire profile of all pathways of SI-3.

**Figure 7:**
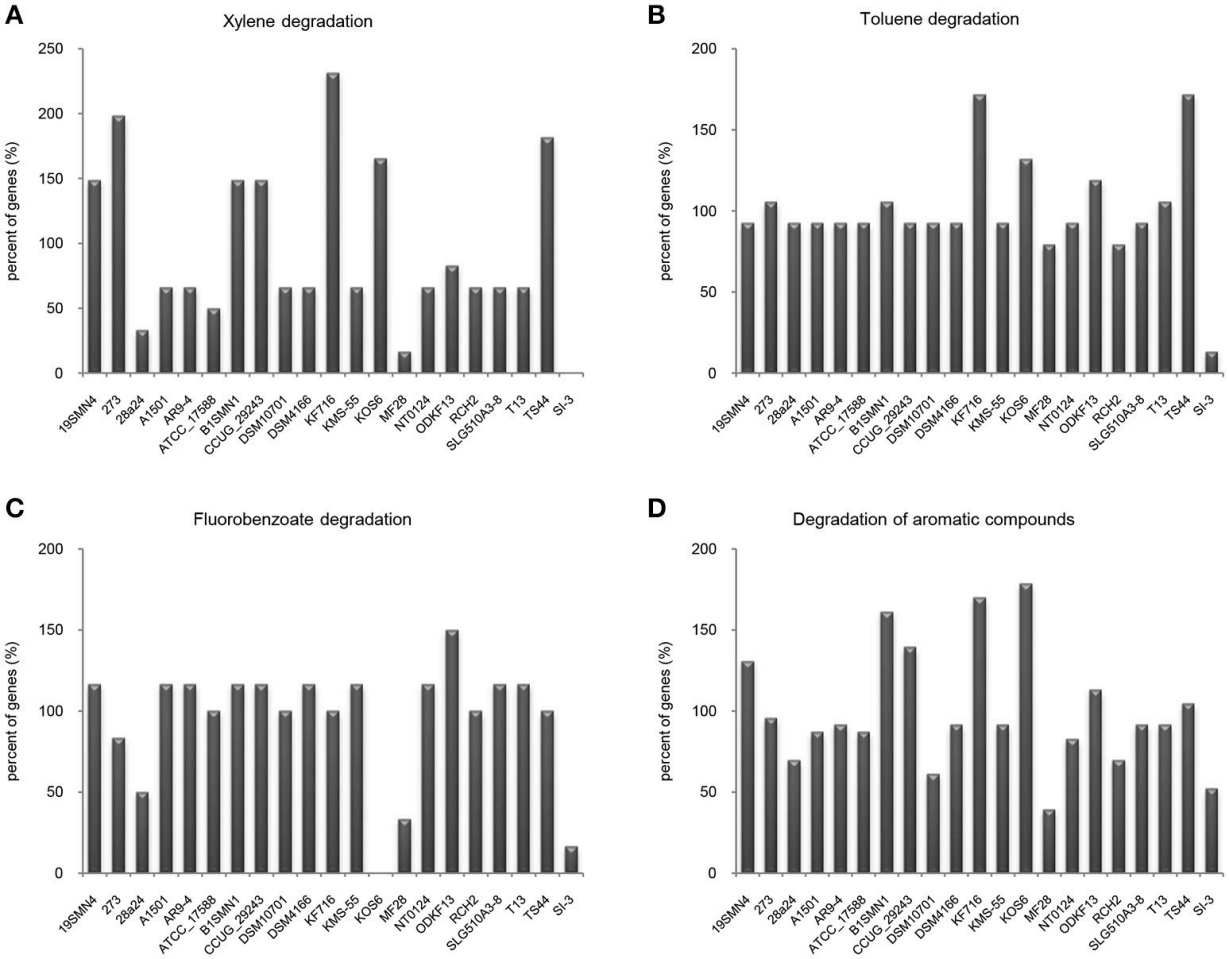
Loss of genes involved in aromatic compounds degradation pathways in *Pseudomonas* sp. strain SI-3. The gene numbers in each strain were normalized to the average gene numbers of SI-3 and 20 *P. stutzeri* strains in xylene degradation **(A)**, toluene degradation **(B)**, fluorobenzoate degradation **(C)**, and degradation aromatic compounds **(D)** pathways.

It is well known that genes work with other genes in a well-organized cooperative relationship rather than functioning individually. To evaluate their biological functions systematically, the overall metabolic pathways of all 21 strains were analyzed according to KEGG analysis. The gene number of each KEGG pathway of strain SI-3 was normalized against the average gene number of the corresponding KEGG pathway of all 21 strains. All KEGG pathways of SI-3 were ranked based on relative gene contents and the gene numbers of most KEGG pathways of SI-3 were close to the averages. Specifically, pathways with relative gene contents lower than 0.8 and higher than 1.2 were presented considering the significant differences compared with the averages (Figure 6). Coincidentally, 8 of 11 metabolic pathways with the relative values lower than 0.8 were involved in degradation of benzene ring containing compounds, indicating the compromised ability of SI-3 to degrade aromatic compounds. The greatest gene loss was observed in pathways of xylene degradation (PATH: ko00622), toluene degradation (PATH: ko00623), fluorobenzoate degradation (PATH: ko00364), and degradation of aromatic compounds (PATH: ko01220). Strain SI-3 was found to be closely related to *P. stutzeri*, which is well known for its metabolic versatility in aromatic compounds degradation (Baggi et al., 1987; Kozlovsky et al., 1993; DiLecce et al., 1997; Dijk et al., 2003; Lalucat et al., 2006), however, there was no xylene degradation pathway in SI-3, indicating the defect of SI-3 in xylene degradation. In addition, the homologs of enzymes catalyzing the initial steps of xylene degradation were absent in strains 28a24 and MF28, which were isolated from soil and oyster, respectively, and closely clustered with SI-3 (Figure 3). In contrast, as the most phylogenetic closely related strain of SI-3, *P. stutzeri* 273 was isolated from a sediment sample collected in the green-tide affected sea area, but the complete set of xylene degradation enzymes was conserved. *P. stutzeri* strains 19SMN4, B1SMN1, CCUG_29243, KF716, KOS6, and TS44 had homologs catalyzing the xylene degradation intermediate methylbenzoate to methylcaltechol (Figure 7A). For the toluene degradation pathway, strain SI-3, 28a24 and MF28 all lacked the enzymes needed to catalyze toluene and its intermediates degradation. Strains KF716, KOS6, and TS44, which were isolated from contaminated environments (Table 1) all contained enzymes responsible for degradation of toluene via 2-hydroxytoluene to 3-methylcatechol. Based on KEGG analysis, strain 273 was able to degrade toluene to benzoate. However, other strains could not degrade toluene, but conserved enzymes catalyzing 4-methylcatechol, the intermediate of toluene-4-sulfonate degradation pathway (Figure 7B). Significant gene loss in fluorobenzoate degradation pathway was observed in strain SI-3, as well as *P. stutzeri* strains 28a24, KOS6, and MF28, all of which lacked the homologs of genes involved in fluorobenzoate degradation (Figure 7C). Hence, the number of SI-3 genes involved in the degradation of aromatic compounds degradation (PATH: ko01220) pathway was rather low (Figure 7D) because the number of genes involved in degradation of multiple benzene ring containing compounds was below average. Collectively, these data, demonstrated that *P. stutzeri* strains isolated from contaminated environments were likely to conserve more pollutant degradation genes, while strain SI-3, a potential endophytic bacterium of *U. prolifera*, lost the majority of genes involved in aromatic compounds degradation. Interestingly, Zhang et al. (2017) demonstrated that sterilized host *U. prolifera* was capable of efficiently removing polycyclic aromatic hydrocarbons. In contrast, enrichment of aromatic compounds biodegradation pathways was found in the rhizoplane microbiome by metagenomic sequencing of root microbes of foxtail millet (Jin et al., 2017). Therefore, we speculated that the bacterial spatial distribution and host capacity of aromatic compounds degradation could shape the adaptation features of the plant-associated bacterial genome.

### Co-existence of two competing dissimilatory nitrate reduction pathways in SI-3

*Pseudomonas stutzeri* is renowned for its specific metabolic properties in nitrogen metabolism, such as nitrogen fixation and denitrification. Since SI-3 was phylogenetically close to *P. stutzeri*, we evaluated the nitrogen metabolism pathways of SI-3 based on KEGG analysis. There were no homologs of *nif* genes, the nitrogen fixation cluster, in strain SI-3. Homologs of nitrogen fixation genes were only identified in *P. stutzeri* strains A1501, DSM4166, KMS55, B1SMN1, and KOS6, among which the first three were all isolated from rhizosphere (Table 1). A complete set of denitrification genes were found in the genome of SI-3, as well as all of the investigated *P. stutzeri* strains except for strain 28a24. Moreover, genes of dissmilatory nitrate reduction to ammonium (DNRA), a competing dissimilatory nitrate reduction pathway of denitrification, were identified in all strains but 28a24. DNRA and denitrification determine fates of nitrate, with the former conserving nitrogen in a more stable and biological preferred form, while the latter serves as a nitrogen sink by turning nitrate into gaseous products. The importance of DNRA in many ecosystems has been ignored until recently (Silver et al., 2001; Rütting et al., 2011). It was proposed that DNRA mechanism by providing ${\text{NH}}_{4}^{+}$ could explain the successive bloom of Texas Brown Tide caused by monospecific microalga *Aureomonas lagunensis* (An and Gardner, 2002). However, the role of DNRA in the bloom of macroalgae has never been considered and studied. Additionally, the interspecific ecological success of *U. prolifera* has been shown to be partially a result of its higher uptake rate of nitrogen (Luo et al., 2012), and ${\text{NH}}_{4}^{+}$ is the preferred nitrogen form (Guo et al., 2017). Since the nitrate concentration of the Yellow Sea presented increasing trend in the last few decades while the concentration of ${\text{NH}}_{4}^{+}$ fluctuated (Li et al., 2015, 2017), we wondered whether coupling of DNRA from associated bacteria with the high nitrogen uptake rate would confer *U. prolifera* a competitive advantages over other seaweeds in the Yellow Sea against other seaweeds. Based on the results of the present study, the role of bacterial DNRA in the algae-bacteria interaction of *U. prolifera* is worthy of further investigation.

### Other factors probably involved in association with *U. prolifera*

#### Substance exchange

Substance exchange is essential to maintenance of an algae-bacteria interaction. *Pseudomonas* strains can use a wide range of nutrients (Clarke, 1982). We identified 118 CAZymes encoded in genome of strain SI-3, including 12 α-amylases (GH13) and five cellulases (GH5) and one β-1,4-xylosidase (GH39), whose substrates were constituents of polysaccharide of *U. prolifera* (Ray, 2006; Lahaye and Robic, 2007). In addition, SI-3 contained 218 genes involved in transport of diverse substrates, such as amino acids, sugars, organic acids, lipopolysaccharide, and biopolymers and 88 genes encoding peptidases or proteases, indicating the capacity of SI-3 to degrade plant-derived compounds. Apart from obtaining organic matter from their hosts, algae-associated bacteria can provide micronutrients in return. For example, iron is essential for most organisms, but often limited in marine environment. The availability of iron regulates the nitrate acquisition in *Ulva* (Viaroli et al., 2005) and utilization of xenosiderophores, high-affinity ferric iron chelators, is a common advantage for cells (Miethke and Marahiel, 2007). Strain SI-3 possessed genes encoding non-ribosomal peptides synthetases involved in the biogenesis of enterobactin, vibrobactin, bacillibactin, and myxochelin precursor according to the analysis against KEGG database, which may contribute to maintenance of its association with *U. prolifera*.

#### Defense

Toxic metals contamination in the margin sea are receiving more and more attention, and numerous studies have shown green macroalgae, such as *U. linza* and *U. prolifera*, are able to absorb and accumulate toxic metals (Jiang et al., 2013). Strain SI-3 contained two different copper resistance systems, *cus* and *cop* (Cha and Cooksey, 1991; Franke et al., 2003), the arsenic-resistance genes *arsC* and*arsRB*, nickel-resistance proteins and multiple toxic metal efflux and transporters. These genes may help SI-3 adapt to high metal niches of its host. In addition to defense against toxic metals, SI-3 also contained many genes involved in defense against oxidative stress, such as oxidative stress response regulator OxyR, catalase and peroxidase. Macroalgae can employ oxidative bursts, in which rapid activation of reactive oxygen species occurs, to defend against pathogens (Goecke et al., 2010). Obviously, the survival and stable existence of endophytic bacteria depend on the ability of these genes to cope with oxidative bursts from their host.

## Conclusion

*Pseudomonas* sp. strain SI-3, which was phylogenetically close to *P. stutzeri*, was isolated from *U. prolifera*, the only dominant alga of the world's largest green tide. Here, we reported the complete genome sequence of strain SI-3 and revealed its unique genomic characteristic as a potential endophyte based on comparison with 20 *P. stutzeri* strains. Stain SI-3 contained more strain-specific genes than most of the others, which may have facilitated its adaptationto the host environment. The remarkably high proportion of genes assigned to transcriptional regulation and signal transduction functional categories indicated the extraordinary ability of SI-3 to respond to its host environment. Loss of genes associated with aromatic compounds degradationwas observed in SI-3, which may be an evolutionary adaptation when associate with its host. Our results revealed the co-existence of DNRA and denitrification, two competing dissimilatory nitrate reduction pathways, in strain SI-3, which was similar to most of the *P. stutzeri* strains it was compared with. The role of bacterial DNRA in algae-bacteria interaction during algal blooming process is noteworthy. Moreover, SI-3 was found to have many genomic traits that probably contributed to maintenance of its algae-bacteria interaction with *U. prolifera*, such as the potential to transport and degrade plant-derived compounds, providing micronutrient to the host and resistance to toxic metals and oxidative stress. Therefore, we suggested that *Pseudomonas* sp. strain SI-3 was a suitable candidate for investigation of the algae-bacteria interaction with macroalgae *U. prolifera*.

## Author contributions

HF designed the study, performed the analysis and interpreted the results. PJ, JZ, and CW contributed to data processing. HF and PJ wrote the manuscript.

### Conflict of interest statement

The authors declare that the research was conducted in the absence of any commercial or financial relationships that could be construed as a potential conflict of interest.

## Supplementary material

Supplementary Video 1 Highly moving bacteria in the thalli of *U. prolifera* observed under optical microscope magnified by 600X. The representative cells in which bacteria existed were indicated by red arrows.

Supplementary Video 2 Highly moving bacteria in protoplast of *U. prolifera* observed under optical microscope magnified by 600X. Several highly moving bacteria were limited within the spherical shape of protoplast all the time. The larger spherical moving particle inside of the protoplast was pushed passively by the bacteria around it.
